# Supplementary material for: Foliar fungal communities strongly differ between habitat patches in a landscape mosaic
Source: PeerJ. 2016 Nov 3;4:e2656. doi: 10.7717/peerj.2656 (PMC5101609; doi:10.7717/peerj.2656)
Supplement: Supplemental Information 2 [file peerj-04-2656-s002.docx]

|  | Reignac | Grand-Verdus | Couhins |
| --- | --- | --- | --- |
| **General characteristics** | | | |
| GPS | N 44°54'03''  W 0°25'01'' | N 44°47'21''  W 0°24'06'' | N 44°45'04''  W 0°33'53'' |
| Sampling dates | 23^rd^ May | 15^th^ May | 22^nd^ May |
|  | 18^th^ July | 16^th^ July | 18^th^ July |
|  | 3^rd^ October | 3^rd^ October | 3^rd^ October |
| **Vineyard characteristics** | | | |
| Grape variety | Cabernet-sauvignon | Cabernet-sauvignon | Merlot |
| Growing method | Conventional | Conventional | Conventional |
| Transect direction from forest edge | South-West | West | North |
| Vineyard row orientation with respect to the edge | Parallel | Perpendicular | Perpendicular |
| **Days between last chemical treatment and sampling (*active substance*)** | | | |
| May | 10 d (*trifloxystrobin, mancozeb, mefenoxam)* | 9 d (*folpel, fosetyl-al, quinoxyfen, myclobutanil)* | 6 d (*metiram, fosetyl-Al, tebuconazole)* |
| July | 7 d (*metiram, cymoxanil, potassium phosphate, sulphur)* | 18 d (*folpel, fosetyl-Al, quinoxyfen, myclobutanil)* | 3 d (*copper, sulphur)* |
| October | 36 d (*copper, deltamethrin)* | 60 d (*copper, zoxamide)* | 10 d (*copper)* |
| **Forest characteristics** | | | |
| Species sampled | Oak, hornbeam | Oak, hornbeam | Oak, chestnut |
| Transect direction from vineyard edge | North-East | East | South |
| **Edge characteristics** | | | |
| Edge width | 5.0 m | 3.5 m | 6.0 m |
