# Supplementary material for: Foliar fungal communities strongly differ between habitat patches in a landscape mosaic
Source: PeerJ. 2016 Nov 3;4:e2656. doi: 10.7717/peerj.2656 (PMC5101609; doi:10.7717/peerj.2656)
Supplement: Supplemental Information 3 — These values were calculated exclusively with dissimilarity between samples within sampling sites (mean ± SD). [file peerj-04-2656-s003.docx]

|  |  |  | May | July | October |
| --- | --- | --- | --- | --- | --- |
| Grapevine | - | Oak | 0.47 ± 0.07 | 0.67 ± 0.09 | 0.91 ± 0.06 |
| Grapevine | - | Hornbeam | 0.50 ± 0.08 | 0.65 ± 0.12 | 0.86 ± 0.06 |
| Grapevine | - | Chestnut | 0.49 ± 0.09 | 0.52 ± 0.08 | 0.92 ± 0.03 |
| Oak | - | Hornbeam | 0.47 ± 0.08 | 0.57 ± 0.11 | 0.72 ± 0.10 |
| Oak | - | Chestnut | 0.45 ± 0.03 | 0.55 ± 0.09 | 0.78 ± 0.09 |
