# Supplementary material for: Foliar fungal communities strongly differ between habitat patches in a landscape mosaic
Source: PeerJ. 2016 Nov 3;4:e2656. doi: 10.7717/peerj.2656 (PMC5101609; doi:10.7717/peerj.2656)
Supplement: Supplemental Information 4 — The response variables are averaged Jaccard dissimilarity matrices, computed over 100 rarefactions of the OTU matrix. In both models, sampling site was included as a stratification variable. The values in bold are significant. [file peerj-04-2656-s004.docx]

|  | F | R² | *P*-value |
| --- | --- | --- | --- |
|  | Foliar fungal community composition | | |
| Date | 4.18 | 0.046 | **0.001** |
| Species | 3.83 | 0.063 | **0.001** |
| Edge | 2.34 | 0.013 | **0.001** |
| D x Sp | 1.98 | 0.065 | **0.001** |
| D x E | 1.22 | 0.013 | **0.020** |
| Sp x E | 1.44 | 0.024 | **0.002** |
| D x Sp x E | 1.03 | 0.034 | 0.232 |
|  | Airborne fungal community composition | | |
| Date | 1.77 | 0.098 | **0.001** |
| Habitat | 1.08 | 0.030 | 0.148 |
| Edge | 0.91 | 0.025 | 0.776 |
| D x H | 0.92 | 0.051 | 0.824 |
| D x E | 0.90 | 0.050 | 0.913 |
| H x E | 1.01 | 0.028 | 0.339 |
| D x H x E | 0.97 | 0.054 | 0.557 |
