# Supplementary figures and images for: Foliar fungal communities strongly differ between habitat patches in a landscape mosaic
Source: PeerJ. 2016 Nov 3;4:e2656. doi: 10.7717/peerj.2656 (PMC5101609; doi:10.7717/peerj.2656)

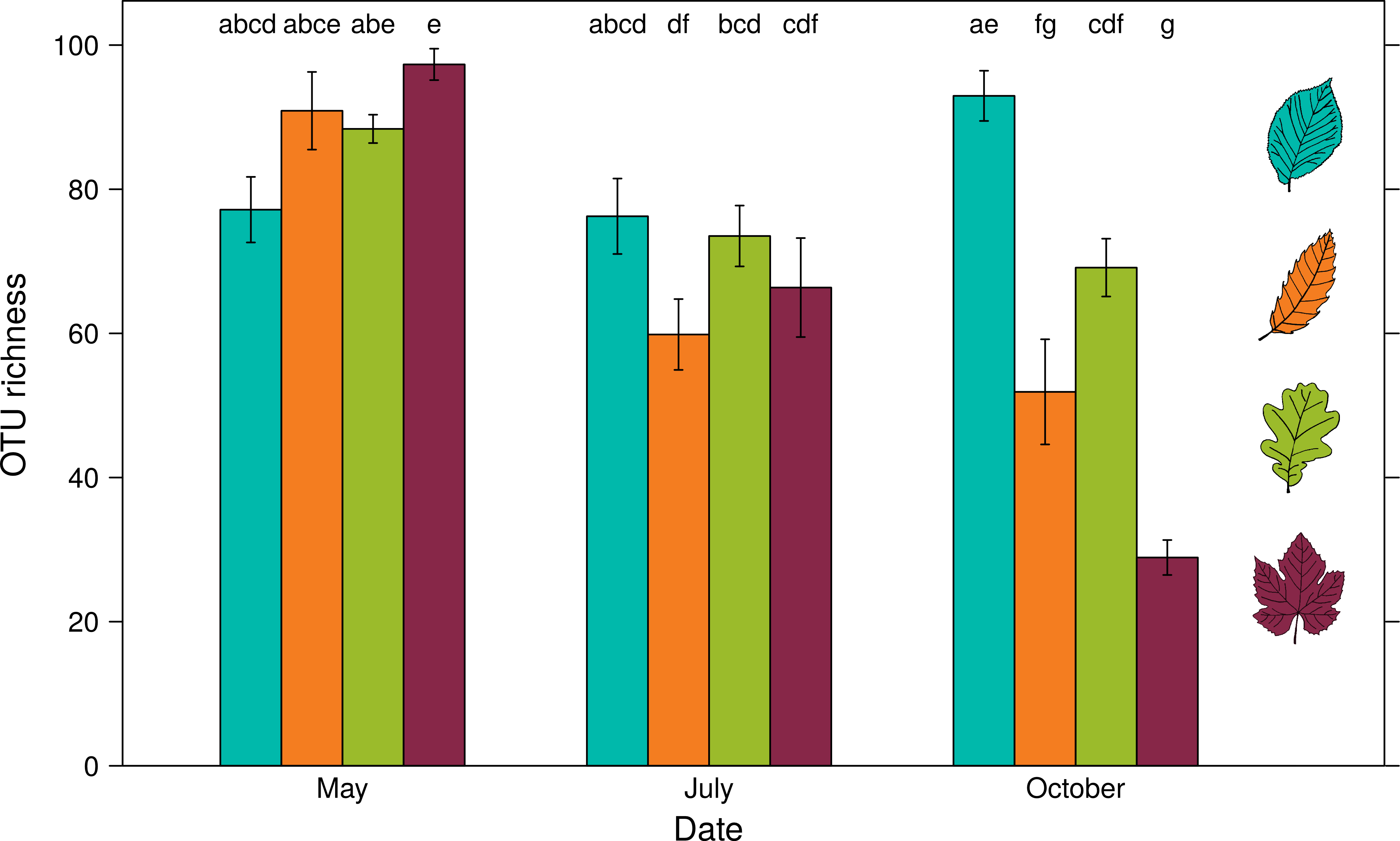

Supplement: Supplemental Information 5 — Oak fungal foliar community richness is represented in green, hornbeam in blue, chestnut in orange and grapevine in red. Error bars represent the SE of the mean. Different letters indicate significant differences in mean (Tukey’s post-hoc pairwise comparison test). [file peerj-04-2656-s005.png]

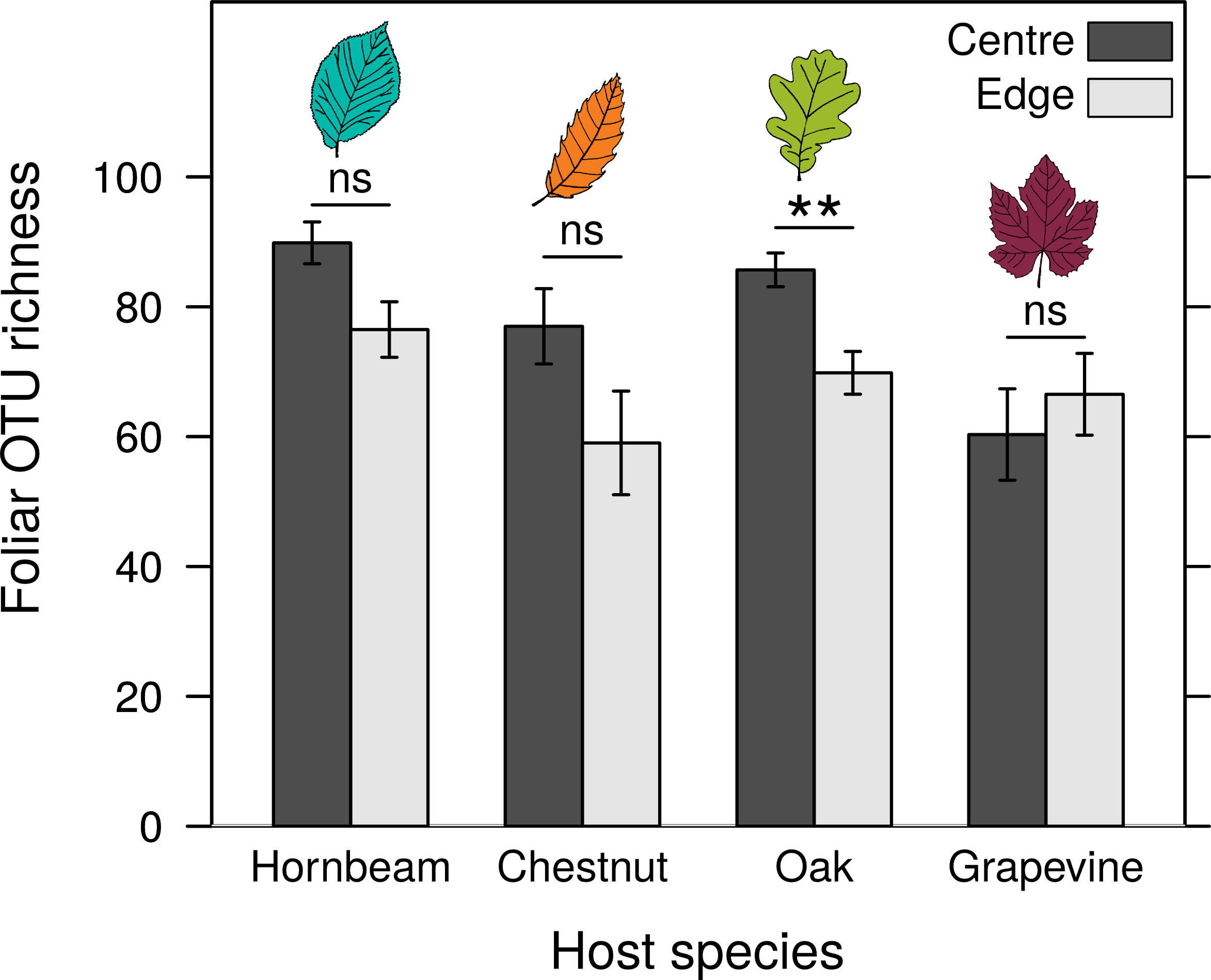

Supplement: Supplemental Information 6 — Error bars represent the SE of the mean. [file peerj-04-2656-s006.png]

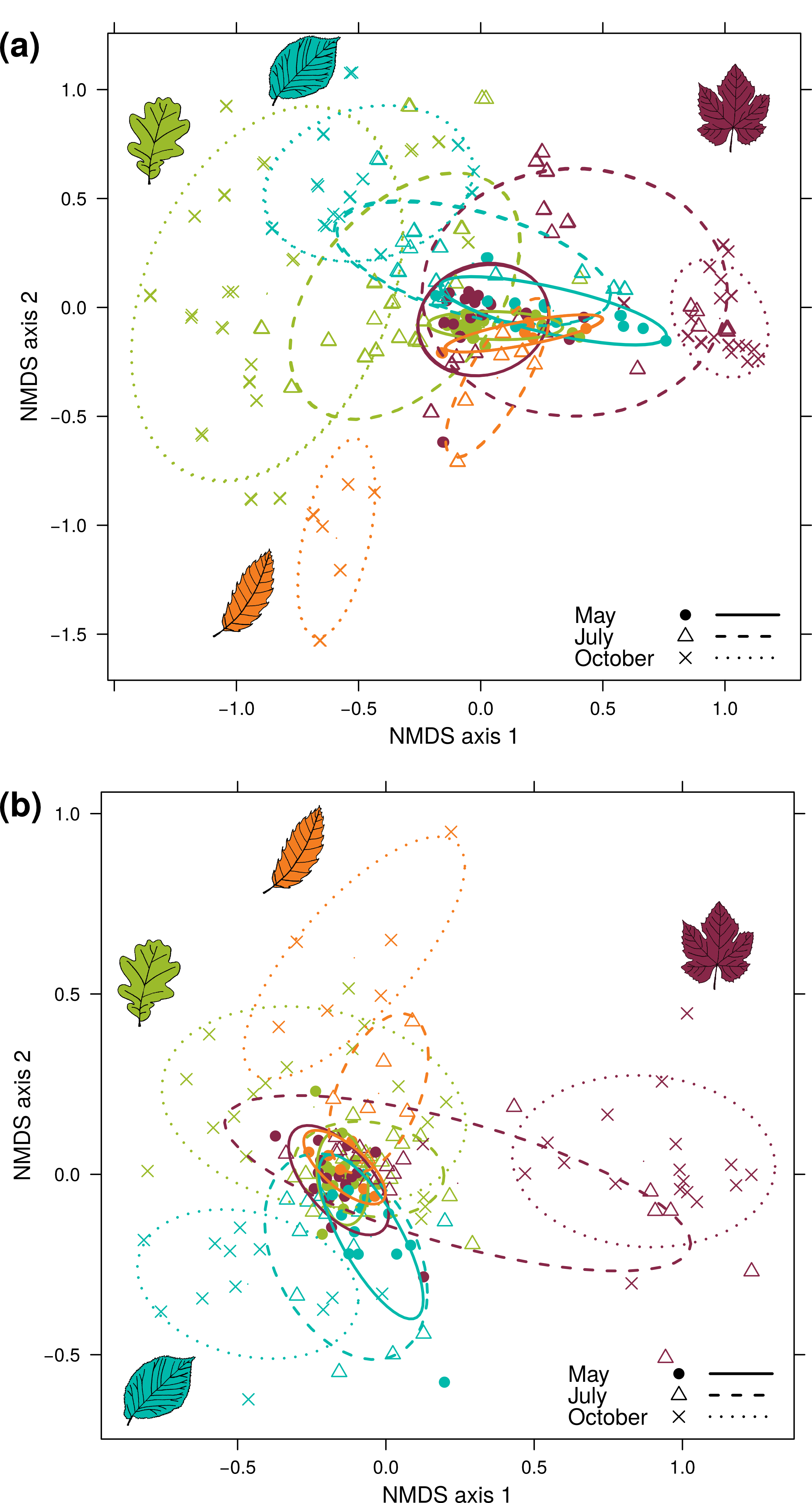

Supplement: Supplemental Information 7 — (A) Bray-Curtis dissimilarities, computed over 100 random rarefaction matrices. The stress value associated with these representations was 0.170. (B) Jaccard dissimilarities, computed over 100 random rarefaction matrices. The stress value associated with these representations was 0.158. Each point represents a sample: colours indicate the host species (hornbeam in blue, chestnut in orange, oak in green and grapevine in red) and the shape of the symbol indicates the sampling date. The confidence ellipsoid at the 0.68 level is shown for all combinations of these two factors. [file peerj-04-2656-s007.png]
