# Supplementary material for: Foliar fungal communities strongly differ between habitat patches in a landscape mosaic
Source: PeerJ. 2016 Nov 3;4:e2656. doi: 10.7717/peerj.2656 (PMC5101609; doi:10.7717/peerj.2656)
Supplement: Supplemental Information 8 — Effect of the abundance (number of sequences per sample), sampling date (May, July or October), host species (oak, hornbeam, chestnut or grapevine) or habitat (vineyard or forest), edge (habitat centre or center) and their interaction on OTU richness in foliar and airborne fungal communities. In both models, sampling site was included as a random variable. Rm2 is the marginal coefficient of determination (for fixed effects) and Rc2 the conditional coefficient of determination (for fixed and random effects). Bold values ares the significant ones. [file peerj-04-2656-s008.docx]

|  | F | *P*-value | R_m_² (R_c_²) |
| --- | --- | --- | --- |
|  | Foliar OTU richness | | |
| Abundance | 57.13 | **<0.001** | 0.62 (0.63) |
| Date | 1.63 | 0.199 |  |
| Species | 12.92 | **<0.001** |  |
| Edge | 11.26 | **0.001** |  |
| D x S | 9.20 | **<0.001** |  |
| D x E | 1.21 | 0.301 |  |
| S x E | 5.56 | **0.001** |  |
| D x S x E | 1.21 | 0.306 |  |
|  | Airborne OTU richness | | |
| Abundance | 28.66 | **<0.001** | 0.57 (0.66) |
| Date | 0.24 | 0.786 |  |
| Habitat | 5.49 | **0.029** |  |
| Edge | 1.23 | 0.279 |  |
| D x H | 0.34 | 0.717 |  |
| D x E | 0.18 | 0.833 |  |
| H x E | 0.08 | 0.780 |  |
| D x H x E | 0.05 | 0.948 |  |
