# Supplementary material for: Foliar fungal communities strongly differ between habitat patches in a landscape mosaic
Source: PeerJ. 2016 Nov 3;4:e2656. doi: 10.7717/peerj.2656 (PMC5101609; doi:10.7717/peerj.2656)
Supplement: Supplemental Information 9 — Effect of sampling date (May, July or October), host species (oak, hornbeam, chestnut or grapevine) or habitat (vineyard or forest), edge (habitat centre or center) and their interaction on the composition of foliar and airborne fungal communities. In both models, sampling site was included as a stratification variable. Bold values ares the significant ones. [file peerj-04-2656-s009.docx]

|  | F | R² | *P*-value |
| --- | --- | --- | --- |
|  | Foliar fungal community composition | | |
| Abundance | 49.21 | 0.177 | **0.001** |
| Date | 6.80 | 0.049 | **0.001** |
| Species | 8.60 | 0.093 | **0.001** |
| Edge | 3.40 | 0.012 | **0.001** |
| D x Sp | 5.78 | 0.125 | **0.001** |
| D x E | 1.73 | 0.012 | **0.022** |
| Sp x E | 2.02 | 0.022 | **0.003** |
| D x Sp x E | 1.08 | 0.023 | 0.266 |
|  | Airborne fungal community composition | | |
| Abundance | 6.49 | 0.157 | **0.001** |
| Date | 1.83 | 0.089 | **0.007** |
| Habitat | 1.43 | 0.035 | 0.086 |
| Edge | 0.79 | 0.019 | 0.696 |
| D x H | 0.72 | 0.035 | 0.895 |
| D x E | 0.80 | 0.039 | 0.808 |
| H x E | 0.97 | 0.023 | 0.418 |
| D x H x E | 0.94 | 0.046 | 0.484 |
